# The impact of craft type on operational spine postures in military boat operators

**DOI:** 10.1016/j.jbiomech.2025.112636

**Published:** 2025-03-16

**Authors:** Joseph A. Gordon, Zachary G. Brumm, Bahar Shahidi, Andrea C. Givens, Brenda A. Niederberger, Emily B. Kloss, Amirali Kamgar, Christian N. Majano, Karen R. Kelly, Samuel R. Ward, David B. Berry

**Affiliations:** a Department of Orthopaedic Surgery, University of California, San Diego, La Jolla, CA, United States; b School of Medicine, University of California, San Diego, La Jolla, CA, United States; c Radiology, University of California, San Diego, La Jolla, CA, United States; d Bioengineering, University of California, San Diego, La Jolla, CA, United States; e Applied Translational Exercise and Metabolic Physiology Team, Warfighter Performance, Naval Health Research Center, San Diego, CA, United States; f Leidos, Inc., San Diego, CA, United States

**Keywords:** Imaging, Musculoskeletal Injury, Spine, Mechanics

## Abstract

High-speed boat operators (HSBO) are exposed to high-impact forces and unstable platforms that are linked to spine pain and musculoskeletal injury risk. This study sought to determine the effects of different military occupational specialties (MOS) on spine kinematics in 86 active-duty personnel (64 HSBO and 22 Marines). The relationships between spine postures, pain, and disability were also examined. Upright MRI scans were performed in sitting and standing positions to determine sagittal cobb angle, angle with respect to the horizontal plane, sacral slope, T1 slope, and intervertebral angles of the lumbar and cervical spine. Disability and pain were assessed with the Oswestry Disability Index (ODI), Neck Disability Index (NDI), and a Visual Analog Scale (VAS). A two-way repeated measures ANOVA analyzed the effects of MOS and position on spine kinematics, and a stepwise linear regression analyzed the influence of pain and disability. Main effects of position were found for lumbar sagittal cobb angle, sacral slope, and intervertebral angles from L2-S1 (p < 0.0001), and cervical sagittal cobb angle (p = 0.02). MOS significantly affected sagittal cobb angle (p = 0.05) and angle w.r.t horizontal (p < 0.0001). Neck disability explained 4 % of the variance in cervical cobb angle, T1 slope, and the intervertebral angle at C5-C6. Pain did not predict lumbar or cervical spine posture. Position has a significant impact on spine kinematics in all groups, with MOS-related differences in cervical spine posture. Subjective pain measures did not reliably predict spine posture, underscoring the necessity for objective diagnostic approaches and targeted interventions to mitigate injury risk in HSBO.

## 1. Introduction

Non-specific back pain is the most prevalent musculoskeletal condition in the U.S. and worldwide, affecting ~ 30 % of individuals at any given time and 80 % of the population at least once in their lifetime (Andersson, 1999; Hoy et al., 2012; Maher et al., 2017; Wu et al., 2020). Spinal pain and injury are critical concerns for military populations due to their economic burden and impact on operational readiness (Bland and Boushey, 1990; Molloy et al., 2020). Military duties impose unique biomechanical stresses on the spine, leading to distinct patterns of spinal posture and increased risk of injury (MacGregor et al., 2012; Mullinax et al., 2023). Despite the role of the cervical spine in stabilizing the neck and rotating the head, far less research has been undertaken in this area when compared to the lower back. Active-duty personnel face greater physical demands than most civilian occupations, leading to higher rates of musculoskeletal (MSK) injuries (Nissen et al., 2014; Yu et al., 2003). These injuries not only reduce deployability but also increase long-term disability costs, highlighting the need for preventive strategies and tailored rehabilitation programs.

Previous studies have identified several positive risk factors for spinal pain and injury in active-duty personnel, such as female gender, time spent wearing body armor, time spent in a motor vehicle, and lower rank (Bader et al., 2018; Knox et al., 2014; To et al., 2021). However, the literature describing the impact of specific occupational demands on the spine remains limited. Previous work has evaluated the effects of load carriage, physical training, and operational position on spine posture in active-duty Marines (Berry et al., 2017; Rodriguez-Soto et al., 2017; Rodríguez-Soto et al., 2017, 2013). These studies assessed the impact of different load distributions and operational postures on spinal alignment, emphasizing the need for preventative measures and targeted interventions tailored to specialized military jobs.

High-speed boat operators (HSBO) routinely experience high-impact forces ranging 4-8G, with peak forces exceeding 20G during maritime transit (Roesch et al., 1994). While other military personnel also encounter impact forces in terrain or aviation settings, the combination of rapid accelerations and decelerations on unstable platforms place unique biomechanical stress on the spine. These conditions contribute to decrements in performance, increased risk of MSK injury, and additional risks such as head jolt and its associated consequences (Myers et al., 2011; Ullman et al., 2022). The HSBO are a military occupational specialty (MOS) that primarily conduct missions in open water conditions on two types of craft: the combatant craft assault (CCA) and the combatant craft medium (CCM). The CCA is a 42-foot vessel that requires operators to remain standing throughout transit. The larger, 60-foot CCM is used for longer duration missions and is equipped with shock mitigating seats, allowing operators to sit during transit. Self-reported data indicate higher rates of lower back pain among CCA operators, attributed to the standing position. Conversely, higher incidences of neck pain have been associated with the unusual neck positions required on the CCM. While standing during high-speed transit is not generally recommended, it may be required due to mission demands. Conversely, seated positions are not without risk, as shock-mitigating seats can bottom out in rough sea conditions, amplifying impact forces transmitted to the body. Though these operational constraints are secondary considerations to mission success, they are likely to contribute to distinct spinal loading patterns and postural adaptations between craft types.

Several studies have underscored the importance of lumbar spine posture for load bearing and trunk stability in military settings (Berry et al., 2018; Cunningham et al., 2010; Kelley et al., 2017). However, the cervical spine plays a critical role in head rotation and neck stabilization, and the impact of occupation on cervical posture has not been evaluated in maritime military personnel. The unique operational positions and high-impact forces experienced by HSBO during transit may have adverse effects on spine postures, leading to chronic alterations in spine mechanics. Therefore, the purpose of this study was to determine the effects of craft type (i.e., CCA vs. CCM) and operational position (i.e., standing vs. sitting) on lumbar and cervical spine kinematics in HSBO. A secondary analysis was performed to compare spine postures in HSBO operators as a function of spine pain and spine-related disability. Using Marines as land-based controls, we hypothesized that operators of CCA would have different lumbar posture than CCM and Marines, and CCM would have a different cervical posture than CCA and Marines.

## 2. Materials & methods

### 2.1. Participants

Eight-six military personnel volunteered to participate in this study, including 64 HSBO (34 CCA and 30 CCM) from the Naval Amphibious Base at Coronado. Additionally, 22 Marines were recruited from the Marine Corps Base Camp Pendleton to serve as land-based controls. All personnel included in analyses were males over 18 years old in an active-duty role. This study was approved by the Institutional Review Boards at the Naval Health Research Center (NHRC#2021.0005) and University of California, San Diego. All participants gave oral and written consent to participate.

### 2.2. Evaluation of spine pain & disability

A visual analog scale, with 0 and 10 cm anchor points (0 = no pain, 10 = worst imaginable pain), was used to assess self-reported pain of the lower back and neck at the time of data collection. Disability was evaluated using the Oswestry Disability Index (ODI) for the lower back and the Neck Disability Index (NDI) for the neck (Fairbank and Pynsent, 2000; Vernon and Mior, 1991). The classification of disability levels for these indices can be found in Table 1 of Spiegel et al. (Spiegel et al., 2016).

### 2.3. Magnetic resonance imaging acquisition

Participants were scanned using an upright 0.6 T magnetic resonance imaging (MRI) scanner (Upright Multi-Position MRI, Fonar Corporation, Melville, NY) using a planar coil. A three-plane localizer (TR = 1254 ms, TE = 100 ms, FOV = 34 cm, matrix = 256 × 256, in-plane resolution = 1.33 mm × 1.33 mm, THK = 9 mm, excitations = 1, time = 0:17) and sagittal T2-weighted fast spin echo images (TR = 1974 ms, TE = 160 ms, FOV = 35 cm, matrix = 224 × 224, in-plane resolution = 1.56 mm × 1.56 mm, THK = 3 mm, gap = 0 mm, NEX = 1, time = 2:12) were acquired.

### 2.4. Operational position

Participants were scanned in sitting and standing positions in order to evaluate spine kinematics. Participants were instructed to assume each position as though they were standing or sitting on the CCA or CCM, and were asked to hold each position for the duration of the MRI acquisition. Photographs depicting exact seating configuration can be found in Fig. 1 of Berry et al. (Berry et al., 2017). Previous research has determined no difference in the test–retest variation in posture (Rodríguez-Soto et al., 2013).

### 2.5. Data analysis

**Fig. 1.**
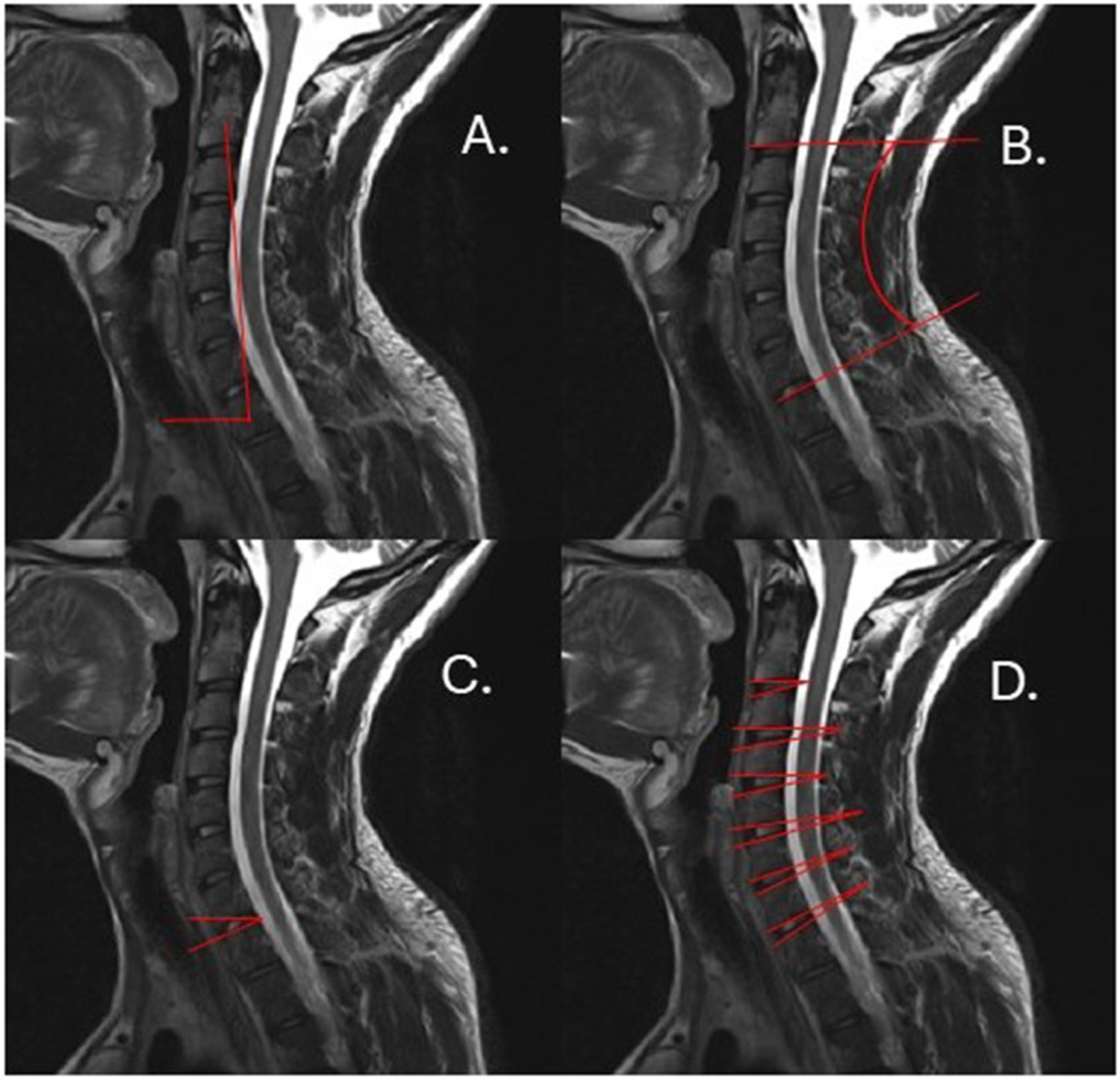


**Fig. 2.**
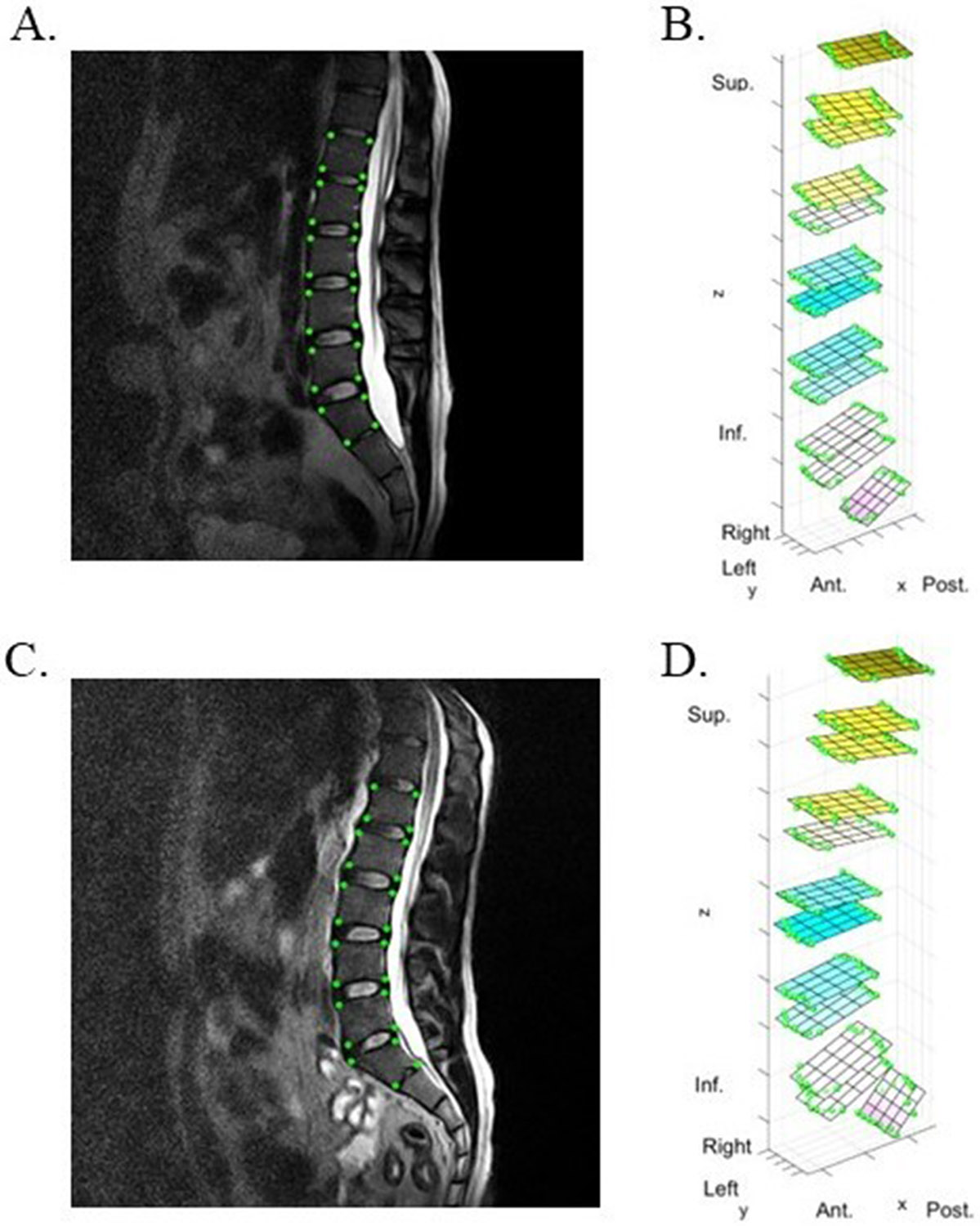


**Fig. 3.**
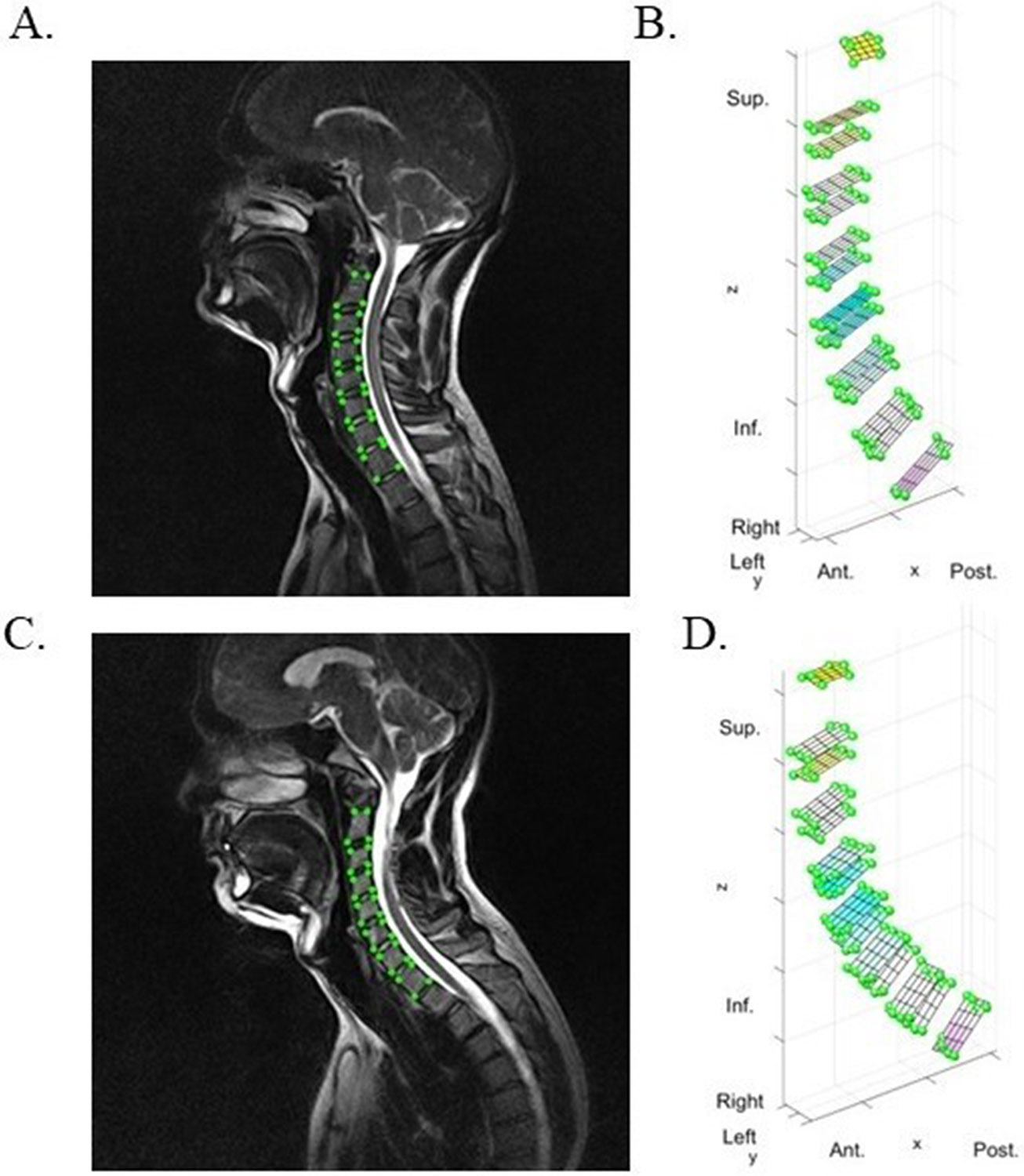


Global and local measurements of lumbar and cervical spine posture were calculated for standing and sitting positions (Fig. 1). Global measurements of sagittal alignment included: angle with respect to (w.r.t.) the horizontal plane, a measurement of forward or backward lean; sagittal cobb angle, a measurement of lordosis or kyphosis; T1 slope, a measurement of sagittal tilt in the cervical spine, and sacral slope, a measurement of sagittal tilt in the lumbar spine. Local measurements included sagittal intervertebral angles, a measurement of lordosis at each vertebral level, and distribution of lordosis throughout the spine. Cervical and lumbar spine kinematics were analyzed from MRI images in each position, as previously described (Berry et al., 2018, 2015). Briefly, digital seed points were manually placed on the corners of the vertebral body from L1-S1 and C2-T1, and on the posterior elements of each vertebra using Horos v.4.0.1. Locations of the seed points were imported into MATLAB (MathWorks Inc., Natick, MA) and used to define an endplate-based joint coordinate system applied to the superior and inferior endplate of each vertebra (L1–S1 and C2-T1). A previous validation study using this technique has shown excellent agreement (ICC = 0.99) between ground truth and measured intervertebral angles (Berry et al., 2015) and low coefficient of variation within user (<0.58°) and between users (<0.90°) for all reported measurements (Rodríguez-Soto et al., 2013). Representative images are depicted in Figs. 2 and 3.

### 2.6. Statistical analysis

All data were tested for normality using the Shapiro-Wilk test prior to analysis. Homogeneity of variances was evaluated using Levene’s test. For all analyses, an alpha level of ≤ 0.05 was used to establish statistical significance. All data are reported as a mean ± SD. Age, height, weight, and body mass index (BMI) were compared using a one-way analysis of variance (ANOVA) to identify differences between the groups (i.e., CCA vs. CCM vs. Marine). A two-way repeated measures ANOVA was performed to analyze the effects of group and position (sitting vs. standing) on lumbar and cervical spine kinematics. This test was selected to account for variability within subjects and between groups. In cases where significant main effects were found, post-hoc pairwise comparisons were performed to identify specific group differences. Sidak tests were used to adjust for multiple comparisons.

To investigate the effect of pain and disability on lumbar and cervical spine kinematics, a stepwise multiple linear regression was performed. All global and local spine kinematic measurements were included as dependent variables. Independent predictors for the lumbar spine included ODI, VAS, and position. Independent predictors for the cervical spine included NDI, VAS, and position. This test was selected to identify the variables that were most predictive of spine kinematics, while limiting the effects of collinearity between these variables. Partial eta-squared effect sizes ($\eta_{p}^{2}$) were reported for ANOVA results, and standardized beta coefficients (β) were reported for regression models. Confidence intervals (95 % CI) were included for effect sizes with significant findings. Statistical analyses were performed using GraphPad Prism version 10.2.2 (GraphPad Software, La Jolla, CA) and SPSS version 28.0 (IBM, Armonk NY).

## 3. Results

Descriptive data for all participants are presented in Table 1. Of the 86 total participants, lumbar images from 5 subjects and cervical images from 2 subjects were excluded due to corrupt or missing data. Marines were significantly older and had more years of service compared to CCA and CCM groups (p < 0.05). Additionally, there were no differences in anthropometric characteristics such as height, body mass, or BMI.

### 3.1. Lumbar posture

**Fig. 4.**
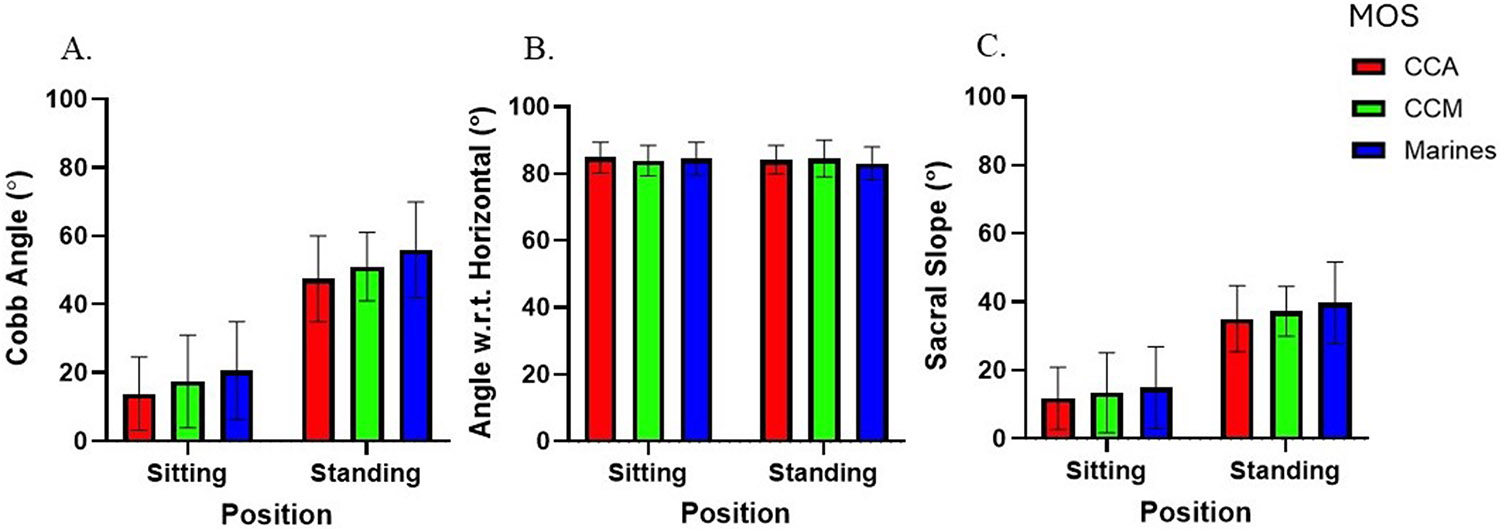


For global lumbar kinematics, there was a main effect of position on sagittal cobb angle (F = 773.8; p < 0.0001; $\eta_{p}^{2}=0.91$, [−36.6,−31.7]) and sacral slope (F = 627.9; p < 0.0001; $\eta_{p}^{2}=0.89$, [−36.63,−31.74]), with greater values in the standing position (Fig. 4). There was no main effect of position on angle w.r.t horizontal (F = 0.57; p = 0.45; $\eta_{p}^{2}=0.007$). No main effects of group were found on sagittal cobb angle (F = 2.89; p = 0.07; $\eta_{p}^{2}=0.24$), angle w.r.t. horizontal (F = 0.21; p = 0.81; $\eta_{p}^{2}=0.005$), or sacral slope (F = 1.07; p = 0.35; $\eta_{p}^{2}=0.03$). There were no significant interactions between position and group on cobb angle (F = 0.21; p = 0.81; $\eta_{p}^{2}=0.006$), angle w.r.t. horizontal (F = 0.97; p = 0.38; $\eta_{p}^{2}=0.49$), or sacral slope (F = 0.19p = 0.83; $\eta_{p}^{2}=0.005$).

**Fig. 5.**
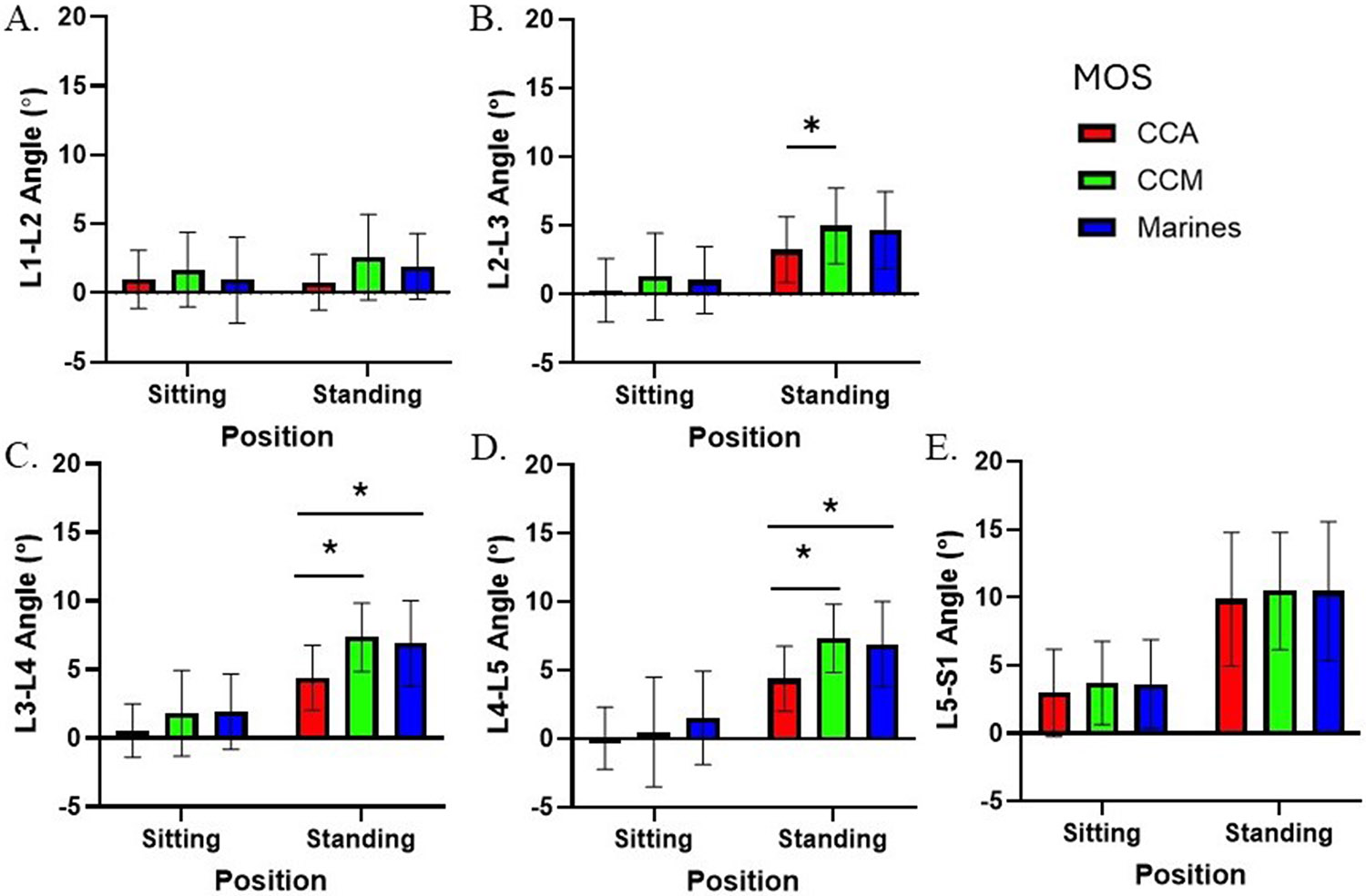


For local lumbar kinematics, there was a main effect of position for the intervertebral angles at L2-L3 (F = 94.9; p < 0.0001; $\eta_{p}^{2}=0.55$, [−4.12,−2.72]), L3-L4 (F = 22.37; p < 0.0001; $\eta_{p}^{2}=0.22$, [−5.41,−4.14]), L4-L5 (F = 241.9; p < 0.0001; $\eta_{p}^{2}=0.76$, [−6.24,−4.83]), and L5-S1 (F = 197.12; p < 0.0001; $\eta_{p}^{2}=0.72$, [−7.80,−5.86]), with greater values in the standing position (Fig. 5). There was no main effect of position at L1-L2 (F = 773.8; p < 0.0001; $\eta_{p}^{2}=0.91$). There was a main effect of group found for intervertebral angles at L2-L3 (F = 3.27; p = 0.04; $\eta_{p}^{2}=0.04$, [0.43,6.31]), L3-L4 (F = 8.20; p = 0.007; $\eta_{p}^{2}=0.10$, [1.56,6.78]), and L4-L5 (F = 5.02; p = 0.008; $\eta_{p}^{2}=0.06$, [0.45,4.92]), with greater values in CCM and Marines compared to CCA in the standing position. There was no main effect of group at L1-L2 (F = 2.56; p = 0.08; $\eta_{p}^{2}=0.03$) or L5-S1 (F = 0.33; p = 0.72; $\eta_{p}^{2}=0.0004$). A significant interaction effect between position and group was observed at L4-L5 (F = 4.79; p = 0.01; $\eta_{p}^{2}=0.06$). There were no significant interactions from L1-L4 or L5-S1(F < 2.87; p > 0.06; $\eta_{p}^{2}=0.07$; all).

### 3.2. Cervical posture

**Fig.6.**
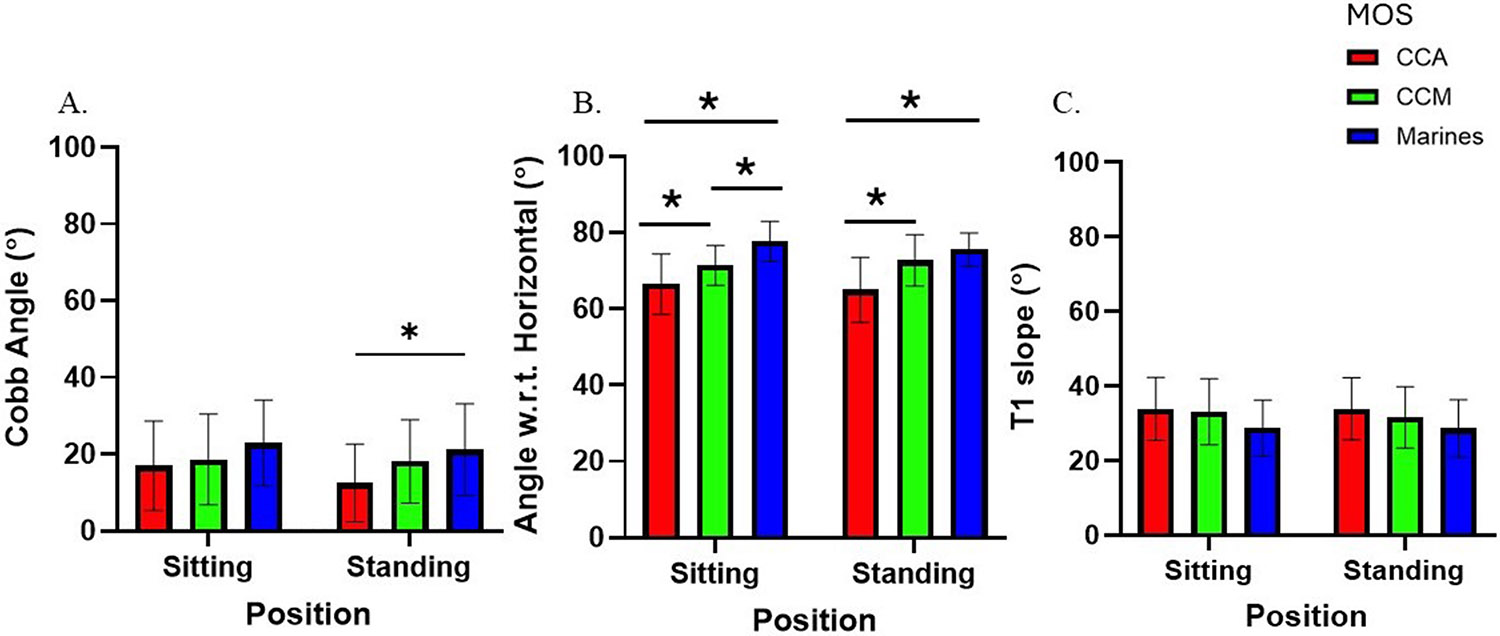


There was a main effect of position on sagittal cobb angle (F = 6.06; p = 0.02; $\eta_{p}^{2}=0.07$, [0.2,0.12]), with greater values in the sitting position (Fig. 6). There were no main effects of position for angle w.r.t horizontal (F = 2.98; p = 0.09; $\eta_{p}^{2}=0.04$) or T1 slope (F = 0.67; p = 0.42; $\eta_{p}^{2}=0.01$). There was a main effect of group on sagittal cobb angle (F = 3.10; p = 0.05; $\eta_{p}^{2}=0.20$, [−16.37,−0.99]), with greater values in Marines when compared to CCA in the standing position. There was a main effect of group on angle w.r.t horizontal (F = 18.92; p < 0.0001; $\eta_{p}^{2}=0.83$, [−5.12, −2.43]), with greater values in Marines and CCM compared to CCA. A significant interaction effect between position and group was observed for angle w.r.t. horizontal (F = 5.51; p = 0.006; $\eta_{p}^{2}=0.12$).

**Fig. 7.**
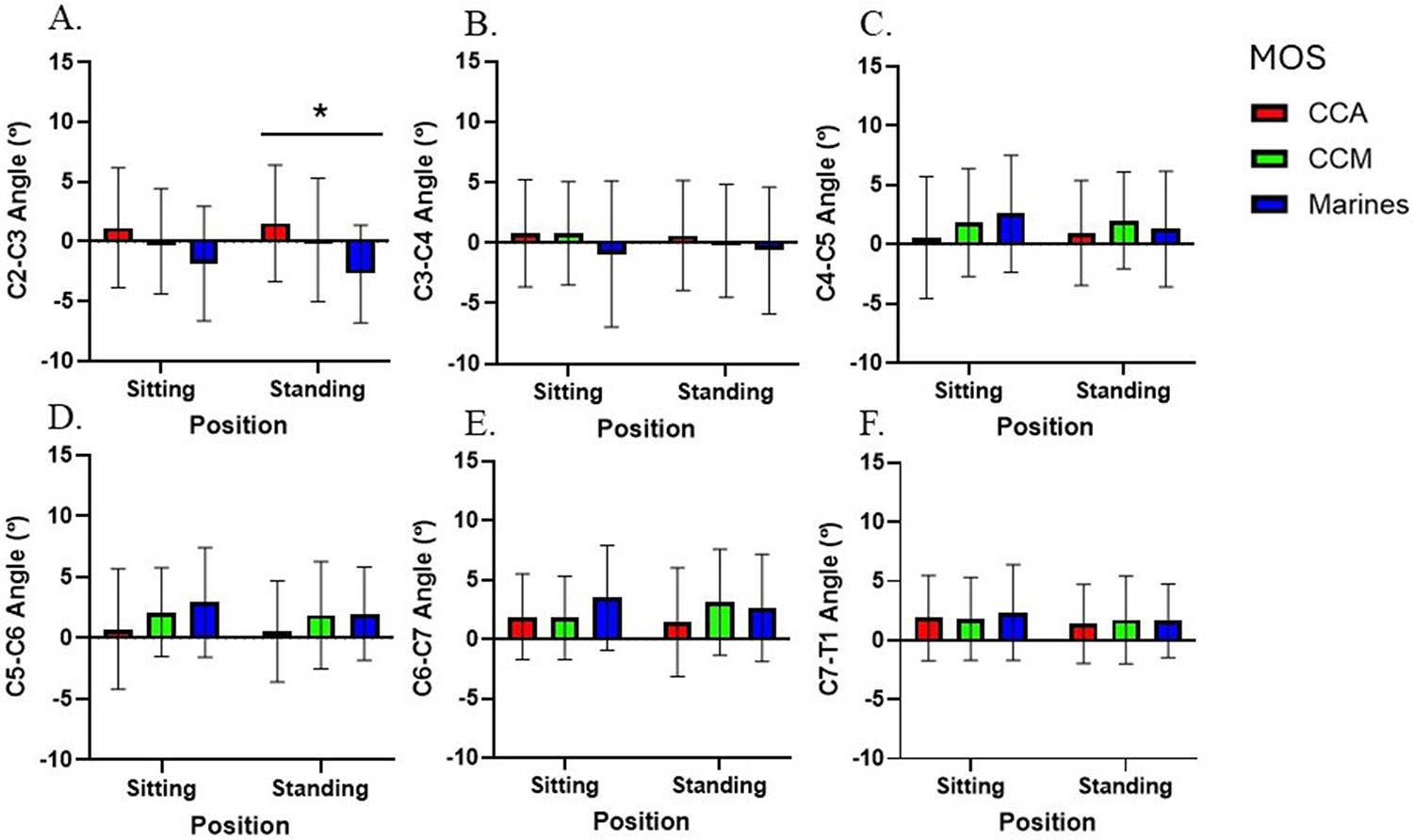


For local cervical kinematics, there were no main effects of position at any intervertebral angle from C2-T1 (F < 1.46; p > 0.23; $\eta_{p}^{2}<0.04$; all) (Fig. 7). There was a main effect of group at C2-C3 (F = 8.09; p = 0.02; $\eta_{p}^{2}=0.16$, [0.98,7.52]), with greater angles in CCA compared to Marines in the standing position. There were no significant interactions from C3-T1 (F < 2.87; p > 0.06; $\eta_{p}^{2}=0.07$; all). There were no significant interaction effects between position and group at any intervertebral angle (F < 1.58; p > 0.76; $\eta_{p}^{2}<0.04$; all).

### 3.3. Pain & disability

For global lumbar kinematics, position explained 65 % of the variance in lumbar cobb angle (β = 0.60) and 58 % of the variance in sacral slope (β = 0.76, p < 0.001; both). For local lumbar kinematics, position explained 29 % of the variance at L2-L3 (β = 0.60), 42 % at L3-L4 (β = 0.65), 61 % at L4-L5 (β = 0.78), and 35 % at L5-S1 (β = 0.59, p < 0.001; all). Neither ODI nor VAS were independent predictors of lumbar spine kinematics.

For global cervical kinematics, NDI explained a small but significant portion of the variance in cervical cobb angle (4 %) (β = −0.20, p = 0.009) and T1 slope (4 %) (β = −0.19, p = 0.012). For local cervical kinematics, NDI explained 4 % of the variance in C5-C6 angle (β = −0.21, p = 0.007). Neither position nor VAS were independent predictors of cervical spine kinematics.

## 4. Discussion

### 4.1. Summary & key findings

In this study, we compared the spine postures of 64 maritime HSBO and 22 land-based Marines to determine the effects of MOS (i.e., sea vs. land operators) and simulated operational positions (i.e., sitting vs. standing) on lumbar and cervical spine kinematics. Additionally, we sought to identify whether validated measures of pain and disability were independent predictors of spine posture in this military cohort. Our key findings highlighted a significant impact of position on spine kinematics, with greater lumbar lordosis and sacral slope in the standing position. Notably, CCA operators exhibited a greater forward leaning cervical posture and reduced lumbar lordosis (i.e., straighter spine) compared to CCM and Marines. These cervical findings in CCA operators may represent an adaptive mechanism for maintaining situational awareness while standing, whereas the reduced lumbar lordosis suggests a postural adaptation to prolonged standing on unstable surfaces. Differences in occupational impact exposure may cause distinct spinal adaptations and variations in injury risk between these groups. Surprisingly, measures of pain were not significant predictors of spine posture. While NDI measures explained a small but statistically significant portion of variance in the cervical spine, three of the four measures of pain and disability utilized did not predict spine kinematics. This disconnect has been reported in other military occupations (Nagai et al., 2014), but not among adults in the general population (Mahmoud et al., 2019). This suggests that posture alone is not sufficient in explaining pain perception or functional impairment in highly trained, specialized populations such as military personnel. Additionally, our sample reported low subjective pain levels, with only 26 % of participants reporting a VAS over 3 in the lower back or neck.

Our hypothesis was partially confirmed. We did not observe differences in global lumbar kinematics for the CCA group compared to CCM or Marines. This may be due to the low anthropometric variability, or high levels of physical training required for all 3 groups. However, there were local differences in lordosis for the lumbar spine between L2-L5 in the CCA group. This is notable, as this may indicate an increased risk of injury in this area of the low back due to decreased curvature of the spine when compared to the other groups. Although some lumbar measurements showed non-significant differences (p = 0.051 to 0.10), these measures may represent early indicators of occupational strain or adaptation. Future research with larger samples and longitudinal designs can help determine whether these trends are biomechanically significant. Our second hypothesis was partially confirmed, as both CCA and CCM had greater forward lean in the cervical spine than Marines while seated. This finding suggests that postural adaptations are the result of MOS rather than anthropometric variation found in our sample. The required standing position for the CCA group may promote forward head posture as a compensatory mechanism to maintain balance or pain reduction. Conversely, the constrained neck movement caused by the seating configurations may lead to changes in cervical alignment in the CCM group when compared to land-based personnel. The various strategies used to optimize performance in HSBO may impose cumulative stresses on spinal structures, accelerating tissue strain, altering load distribution, and increasing injury risk.

### 4.2. Practical Applications & Implications

Back pain is pervasive in both civilian and military populations (Hoy et al., 2012; Knox et al., 2011). While several studies have sought to identify risk factors for spine-area pain in military personnel (Cohen et al., 2012; O’Connor and Marlowe, 1993), few have evaluated the effects of specific occupations on MSK anatomy. Our study found greater lordosis and pelvic tilt in the lumbar spine when standing, regardless of craft type. These findings are consistent with previous research in military personnel (Berry et al., 2017; Rodríguez-Soto et al., 2013) and civilian populations, (Alamin et al., 2018; Bridger et al., 1989) indicating that changes in position (sitting vs. standing) affect lumbar spine alignment. Our findings also demonstrated pronounced group-related differences in neck posture, despite no significant associations between age, BMI, or years of service with spine kinematics. We would expect service members of older age and more years of service to have adverse postural adaptations such as forward head posture, due to cumulative exposure to occupational stressors. This suggests that the demands of maritime transit (e.g., slamming impacts, unstable platforms) are stronger drivers of postural adaptations than anthropometric factors alone. This study adds to the literature by utilizing a unique cohort of maritime military personnel. Studies often recruit civilians to replicate military tasks or attempt to apply findings from research in non-military populations to military cohorts. However, service members undergo specific training, wear specialized gear (e.g., body armor, night vision devices, helmets), and have higher exposure to injury; all factors that fundamentally differentiate their biomechanical responses from the general population. Our findings show differences in neck posture in the absence of significant pain, which highlights the need for a multimodal approach that integrates subjective report, objective measurement, and task-specific assessment to accurately evaluate job-related changes or injury risk.

Our study found no relationship between self-reported pain and spine posture. This underscores the importance of objective, applicable assessment of spinal health, which is a key risk factor for operational availability. The natural lordotic shape of the cervical and lumbar spine supports the body by distributing mechanical stress, thereby minimizing strain on individual vertebrae, intervertebral discs, and associated soft tissues. Quantifying the job-related changes can clarify the structure–function relationship in groups with specialized roles, helping researchers identify populations at the greatest risk for injury before operational duties and evaluate the effectiveness of pain management strategies. A recent review by Ullman and colleagues reported a three-fold greater risk of injury in HSBO compared to all other special operators, and a 43-fold greater risk compared to the general working population (Ullman et al., 2024). The lower back (26 %) and neck (16 %) were the most injured locations in HSBO. The lack of a neutral neck position may contribute to the smaller degrees of lordosis (i.e., straighter spine) and the increased forward lean observed in the CCA group, which could be a mechanism of injury for boat operators without neck support. Additionally, the CCA and CCM groups exhibited more cervical forward lean than Marines in their respective operational positions. This data may help us explain why clinical measures of pain and disability are uncoupled from anatomical measurements. These findings lay the groundwork for investigating the mechanisms of maladaptive spinal alterations in HSBO, and provide insight into the subclinical tissue changes that may precede injury. Paraspinal muscle strength and core stability are essential for maintaining an upright position (Hodges and Richardson, 1996; Panjabi, 1992). Therefore, future evaluation of strength and endurance in these postural muscles are worthy of further investigation to mitigate injury risk from prolonged standing, unstable platforms, and whole-body slamming impacts (To et al., 2021; Ullman et al., 2022).

### 4.3. Limitations and Future Directions

This study has several limitations that offer opportunities for future work. One limitation includes the number of subjects included in each group. There are only 400 HSBO in the United States, approximately 15 % of which were recruited for this study. A *post hoc* power analysis indicated sufficient power to detect moderate (f = 0.25; α = 0.05; power = 0.95) and large (f = 0.40; α = 0.05; power = 0.99) differences between groups, but not small (f = 0.02; α = 0.05; power = 0.16) ones. Despite the fact that our study is sufficiently powered to identify meaningful relationships between predictors and outcomes, it is important to recognize that more subtle differences may be missed due to sample size limitations. Additionally, given the widespread nature of back pain, some reported pain or injury may be unrelated to job duties. This study did not directly account for potential confounders such as prior injury, training intensity, or cumulative operational exposure, which may influence spine kinematics (Berry et al., 2017). However, detailed analysis of these factors was not feasible due to sample size constraints. The cross-sectional design of this study also prevents us from establishing a causal link between MOS and spine posture. It is possible that participants may have had underlying pathologies that contributed to their posture independently of job-related activities or symptoms. However, previous work has shown short-term decrements in physical performance following a high-speed boat ride (de Alwis et al., 2021; Myers et al., 2011), therefore it is plausible to expect occupation-related changes in pain or posture. Another limitation is the inability to evaluate the whole-spine in a single acquisition, due to limited field-of-view. This prevents assessment of center of gravity or regional spine interactions. Future research should incorporate advanced imaging techniques and multiparametric outcomes related to MSK physiology, such as disc health and muscle composition, to improve our understanding of the changes that result from operating high-speed maritime vessels. Longitudinal studies should investigate changes in spinal posture and functional performance at critical timepoints such as operator training, deployment workup, deployment, and post deployment to clarify the long-term effects of HSBO duties.

### 4.4. Conclusion

This study provides valuable insights into the spinal kinematics of HSBO, highlighting the significant impact of operational positions on both lumbar and cervical spine alignment. Our findings reveal group-related differences in the cervical spine for HSBO when compared to Marines. Subjective measures of pain were not related to spine postures. However, measures of neck disability had a small but significant relationship with cervical spine kinematics. These data are important in identifying the measurable changes in the spine that may or may not be associated with pain and injury in this unique MOS. In the long term, this information can be used to develop biomechanical models that can quantify the effects of job type or mission duration on MSK health and function. In short, future studies can provide a more comprehensive understanding of the long-term impacts of military occupational demands on spinal health, enhancing the health and operational readiness of military personnel.

## 5 Disclaimer.

I am a military service member or employee of the U.S. Government. This work was prepared as part of my official duties. Title 17, U.S.C. §105 provides that copyright protection under this title is not available for any work of the US Government. Title 17, U.S.C. §101 defines US Government work as work prepared by a military service member or employee of the US Government as part of that person’s official duties. Report No. 24–75 was supported by the Defense Health Agency under work unit no. N2024. The views expressed in this work are those of the authors and do not necessarily reflect the official policy or position of the Department of the Navy, Department of Defense, or the US Government. The study protocol was approved by the Naval Health Research Center Institutional Review Board in compliance with all applicable Federal regulations governing the protection of human subjects. Research data were derived from an approved Naval Health Research Center Institutional Review Board protocol, number NHRC.2021.0005.

## Figures and Tables

**Table 1:** Subject Characteristics and Spinal Health Measures for Cervical and Lumbar Spine.

	Cervical			Lumbar		
	CCA (n = 33)	CCM (n = 31)	Marines (n = 20)	CCA (n = 30)	CCM (n = 30)	Marines (n = 21)
Age (years)	26.6 ± 5.7	27.6 ± 5.3	31.2 ± 4.6	26.8 ± 5.9*	27.5 ± 5.3*	30.9 ± 4.6
Height (cm)	178.6 ± 6.2	178.5 ± 6.3	177.3 ± 7.2	178.0 ± 6.0	178.6 ± 6.3	178.0 ± 7.8
Weight (kg)	86.5 ± 9.2	85.9 ± 11.1	85.6 ± 10.4	85.6 ± 8.6	85.9 ± 11.3	85.5 ± 10.2
BMI (kg·m^−2^)	27.1 ± 2.5	27.0 ± 2.9	27.2 ± 2.6	27.0 ± 2.4	27.0 ± 2.9	27.0 ± 2.8
Years of Service	5.5 ± 5.4*	6.1 ± 5.3*	11.6 ± 4.7	5.6 ± 5.5*	6.0 ± 5.4*	11.3 ± 4.7
NDI or ODI	9.0 ± 12.5	9.5 ± 11.5	10.4 ± 11.7	15.6 ± 15.2	19.9 ± 18.4	20.4 ± 11.6
VAS	1.0 ± 1.3	1.5 ± 1.9	1.1 ± 1.8	1.2 ± 1.6	1.7 ± 1.8	1.8 ± 1.9

* Indicates significant differences (p < 0.05) compared to Marines.
